# Neutrophil Gelatinase-Associated Lipocalin (NGAL) as a Predictor for Sepsis Mortality in Children Admitted to Pediatric Intensive Care Unit (PICU): A Comparison With Prothrombin Time/International Normalized Ratio (PT/INR) and Urea/Creatinine Ratio

**DOI:** 10.7759/cureus.72643

**Published:** 2024-10-29

**Authors:** Marwa Ibrahem Abdelrazic, Gehan Lotfy Abdel Hakeem, Mina Sobhy Hanna, Omima M Mohamed, Doaa Elzaeem Ismail, Ibtehal Saad Abuelela

**Affiliations:** 1 Pediatrics, Faculty of Medicine, Minia University, Minya, EGY; 2 Clinical Pathology, Faculty of Medicine, Minia University, Minya, EGY

**Keywords:** children, pt/inr ratio, sepsis, serum neutrophil gelatinase associated lipocalin, urea/creatinine ratio

## Abstract

Background

Sepsis is the primary cause of death in children, and it is crucial to identify patients at high risk of mortality early on in order to provide intensive monitoring and management in the Pediatric Intensive Care Unit (PICU).

Objective

The objective of this study was to assess the predictive value of routinely used sepsis indicators, including neutrophil gelatinase-associated lipocalin (NGAL), urea to creatinine ratio (urea/Cr), and prothrombin time and international normalized ratio (PT/INR), in predicting death in critically unwell children.

Patients and methods

A total of 75 children were included in the research conducted at the PICU of Minia University. Among them, 21 (28%) were released as survivors, while the remaining 54 (72%) unfortunately passed away. All participating children were subjected to serum NGAL, urea/Cr, and PT/INR measurements during the first 24 hours of hospitalization. The severity of sepsis was assessed using the Pediatric Risk of Mortality (PRISM) III score.

Results

The NGAL, prothrombin, urea, and creatinine levels were considerably elevated in the group of individuals who died compared to those who survived (P < 0.001, 0.007, 0.028, and 0.032, respectively). However, no significant difference was found between survivors and deceased children in terms of the PT/INR ratio and urea/Cr ratio. When predicting mortality, NGAL with a cutoff point of more than 990 had a sensitivity of 100% and a specificity of 35%. Similarly, the PRISM score with a cutoff point greater than 18 had a sensitivity of 83.3% and a specificity of 42.9%.

Conclusion

Serum NGAL is reliable in the early prediction of mortality in children admitted with sepsis.

## Introduction

Sepsis is a life-threatening condition that has an impact on the biochemistry and physiology of the body [1]. About 49 million people are affected by sepsis each year, and it is believed to be the cause of over 11 million deaths globally, accounting for up to 19.7 percent of all fatalities [2].

Although up to 25% of individuals still die from sepsis, overall death rates seem to be decreasing. Septic shock is associated with a mortality rate of over 60% in hospitals [3]. As many as 80% of pediatric fatalities are attributed to sepsis [4]. Care during the early stages of sepsis, including rapid diagnosis and precise categorization, is crucial for reducing mortality [5].

While clinical scoring methods are helpful in determining the severity and prognosis of sepsis, they often take 12 to 24 hours to finish. The discovery of biomarkers for early identification and evaluation of sepsis prognosis is a time-consuming yet very important endeavor [6]. There is evidence that shows an increase in neutrophil gelatinase-associated lipocalin (NGAL) levels when infection is present [7]. A member of the lipocalin family, NGAL is a 25 kDa protein [8].

Multiple studies have shown a correlation between plasma NGAL and the immune response; moreover, a strong correlation was found between plasma NGAL and the production of interleukin 6 (IL-6), interleukin 10 (IL-10), and tumor necrosis factor-alpha (TNFα), suggesting that plasma NGAL may be associated with inflammation [9]. Prior studies found that NGAL serum levels were greater in patients with septic illnesses [10].

Up to 80% of patients with septic shock will have coagulopathies, a frequent consequence of sepsis [11]. Uncontrolled activation of the clotting cascade may cause intravascular thrombosis and disseminated intravascular coagulation (DIC), which can lead to hemorrhagic and potentially deadly thrombotic episodes. According to research, severely sick individuals with DIC had a 28-day death rate of 20% to 50% higher than those without the condition [12]. It is very desirable to find better ways to detect sepsis-associated coagulopathies and develop new ways to treat them. In a recent study, it was shown that sepsis survivors had lower activated partial thromboplastin time (aPTT) levels than non-survivors. This finding implies that the prothrombin time and international normalized ratio (PT/INR), which is a standardized tool for labs and reagents, may provide more accurate diagnostic and prognostic information for sepsis patients [13].

The levels of creatinine (Cr) and blood urea may indicate the extent to which the kidney's glomerular filtration function has been compromised by environmental variables. It is usual for urea levels to rise in cases of gastrointestinal bleeding and other medical conditions. An increase in red blood cell (RBC) production and the absorption of plasma proteins as a source of nitrogen may occur after intestinal hemorrhage [14]. When assessing renal function in a clinical setting, Cr content is a common tool for spotting variations that could indicate a possible failure or an improved condition of renal function. The levels of urea and Cr are affected in several circumstances [15,16]. Predictions based on urea or Cr alone may not be comprehensive since urea is not a particular marker of renal insufficiency. There is new evidence that the urea to creatinine (urea/Cr) ratio is a prognostic factor for individuals with acute decompensated heart failure, acute cerebral infarction, acute renal damage, and ischemic stroke [17-20]. The correlation between urea/Cr and septic shock prognosis has been the subject of few investigations, nevertheless.

This study aimed to evaluate the serum NGAL levels in children admitted to the Pediatric Intensive Care Unit (PICU) with sepsis or septic shock, as well as compare and correlate this level with the clinical course and mortality outcome in critically ill children. Additionally, it aimed to assess the prognostic role of the PT/INR and the urea/Cr ratio on mortality.

## Materials and methods

Study methodology and environment

Minia University Children's Hospital served as the site of this prospective hospital-based research, which was conducted from September 2022 to March 2023. All children whose parents or legal guardians gave their written permission after receiving a thorough explanation of the research were selected.

Patients' selection

The study included 75 children who were hospitalized with sepsis in the PICU of Minia University Children’s Hospital. Eligible patients were of ages between one month and 12 years.

Inclusion Criteria 

1. Age between one month and 12 years old 2. Admitted to PICU 3. Diagnosed with sepsis according to the International Consensus Conference on Pediatric Sepsis. Two or more criteria for systemic inflammatory response syndrome, confirmed or suspected invasive infection, and cardiovascular dysfunction, acute respiratory distress syndrome, or two or more organ dysfunctions were used by the International Consensus Conference on Pediatric Sepsis to diagnose sepsis in patients [21]. Furthermore, sepsis was a diagnosis made upon admission for all patients included in the research. Several criteria were used to diagnose septicemia: (i) A body temperature that is either higher than 38.5°C or lower than 38.5°C (the axillary method was used to measure temperature); (ii) a faster heart rate; (iii) a faster respiratory rate; (iv) a leukocyte count that is either too high or too low for the patient's age, or 10% immature neutrophils; and (v) a positive blood culture in addition to the main illness symptoms. The presence of any of the following clinical indications of inadequate tissue perfusion was used to diagnose septic shock: (i) a diminished or altered mental state; (ii) capillary refilling that lasts more than two seconds; (iii) weaker pulses; (iv) cold extremities that are mottled; (v) urine output of less than 1 ml/kg/hour; and (vi) persistent hypotension despite adequate fluid intake [22]. The symptoms of septicemia are also present. 4. Informed written consent had been obtained from their caregivers

Exclusion Criteria

1. Children neonates (children under 28 days of age) 2. Children with hematologic diseases (including hematological malignancies and immunodeficiency diseases) 3. Children with a hospital stay of fewer than three days 4. Children with incomplete clinical data 5. Children without parental consent

Collecting data

Baseline demographics, PICU admission diagnosis, complications (including septic shock and multi-organ failure), and all factors necessary to compute the Pediatric Risk of Mortality (PRISM) III score were all part of the data gathered. A PRISM III calculator, which can be accessed online at www.medal.org, was used to enter the data [23]. The following is the standard way to interpret the PRISM III scores: 0-5, 6-10, 11-15, 16-20, 21-25, and 26-30. The corresponding mortality rates are 11%, 23%, 40%, 61%, 78%, and 89% [23].

Evaluations based on blood samples

The intensive care unit frequently used vacutainer equipment to collect all blood samples, including prothrombin time (PT), urea, Cr, and NGAL, from a venous line. In the first 24 hours after admission to the PICU and before the commencement of antibiotic treatment, 5 ml of venous blood was aseptically taken from each patient group [24].

To calculate PT and the PT/INR ratio, 1.8 ml of blood was mixed with 0.2 ml of 0.2% trisodium citrate in a test tube. Subsequently, urea and Cr were determined by centrifuging the sodium citrate tube and analyzing the resulting plasma. The last 3 ml were transferred to an empty tube, allowed to clot, and then spun in a centrifuge. In preparation for the NGAL test, the expressed serum was flash-frozen at -70 °C [25]. Blood samples collected 90 minutes after being admitted to the intensive care unit were not included.

Methods

The hospital's certified laboratory conducted all laboratory tests. The turbo-densitometric technique was used to measure PT/INR. The LABiTec PT-Reagent kit and LABiTec coaDATA 4004 were purchased from Biomedical Technologies GmbH, Ahrensburg, Germany. The procedure was carried out in accordance with the manufacturer's instructions. The typical range for PT/INR is less than 1.2 [26]. Auto-analyzer SELECTRA PRO XL (ELITech Group, Clinical Chemistry Automation Systems, Amsterdam, Netherlands) was used to assess blood urea levels and serum Cr. Commercially available kits were used in accordance with the manufacturer's instructions. For serum NGAL evaluation, the levels of serum NGAL were measured using an authorized commercial dual monoclonal antibody (MA) sandwich immunoassay kit (Human Neutrophil Gelatinase-Associated Lipocalin ELISA Kit; Glory Bioscience, Del Rio, Texas, USA) [27].

Ethical approval

This study was approved by the ethics committee (approval no: 388:2022, dated 19 September 2022). Prior to participating in the research, every parent signed a formal consent form.

Statistical study

For the statistical study, we used the IBM SPSS Statistics for Windows, Version 26.0 (IBM Corp., Armonk, USA). Using the Kolmogorov-Smirnov tests, we checked whether the data was normally distributed. For quantitative measurements, the data was presented as mean and standard deviation. For classified data, the lowest and maximum ranges were used. In order to compare the two separate groups, the Mann-Whitney U test for non-parametric data was used. Two distinct groups were compared using the chi-square test. The association between biomarkers and sepsis mortality was examined using simple logistic regression and multiple stepwise regression analyses. Using receiver operator characteristic (ROC) and area under the curve (AUC), we evaluated the predictive efficacy of biomarkers for sepsis mortality. For statistical significance, a p-value of less than 0.05 was considered.

## Results

The 75 children hospitalized in the PICU at Minia University Children's Hospital with sepsis between September 2022 and March 2023 were part of this prospective hospital-based research. There was no statistically significant difference in age or sex between the two groups; however, multi-organ failure was more common in the non-survivors (38.9% vs. 19%) than in the survivors (28%). Twenty-one of these patients were released, making them survivors. The other 54 patients, or 72.2%, were non-survivors. 

Significant variations in the PT, urea, Cr, and NGAL levels were observed between the non-survivor and survivor groups with respect to the examined indicators as shown in Tables 1-2.

**Table 1:** Comparison between non-survivors and survivors regarding baseline clinical characteristics N: Number of cases; PRISM III: Pediatric Risk of Mortality III Score, a scale used to predict the mortality rate of children admitted to intensive care units; p-value: Represents the level of statistical significance, where * indicates statistical significance (usually P<0.05 indicates a statistically significant difference between the groups).

Sociodemographic Data	Non-Survivors (N=54)	Survivors (N=21)	p-value
Age (years): Mean ± SD	5.42 ± 3.04	5.63 ± 3.21	0.809
Sex N (%): Male	25 (46.3%)	11 (52.4%)	0.561
Severe Sepsis N (%):	18 (33.3%)	7 (33.3%)	0.004*
Multi-organ Failure N (%):	21 (38.9%)	4 (19%)	0.004*
PRISM III: Mean ± SD	24.7 ± 6.65	21.3 ± 6.91	0.055

**Table 2:** Comparison of laboratory markers between non-survivors and survivors PT: Prothrombin time; PT/INR: Prothrombin time/international normalized ratio; Cr: Creatinine; NGAL: Neutrophil gelatinase-associated lipocalin; p-value: Indicates the statistical significance level, where * denotes a significant p-value (usually P<0.05).

Laboratory Markers	Non-Survivors (N=54)	Survivors (N=21)	p-value
PT: Mean ± SD	16.8 ± 4.86	14.5 ± 2.12	0.007*
PT/INR: Mean ± SD	1.36 ± 0.43	1.15 ± 0.2	0.065
Urea: Mean ± SD	89.8 ± 23.1	76.8 ± 21	0.028*
Cr: Mean ± SD	1.77 ± 1.06	1.24 ± 0.37	0.032*
Urea/Cr Ratio: Mean ± SD	58.1 ± 20	63.8 ± 16.2	0.253
NGAL: Mean ± SD	1017.9 ± 574	642.5 ± 148	<0.001*

Tables 3-4 show the results of a simple logistic regression analysis that looked at the examined biomarkers and other factors for their ability to predict death. The results showed that INR, urea, Cr, and NGAL were significant predictors of mortality. In Table 5, we can see that after further examination of the same variables using a multiple stepwise logistic regression analysis for mortality prediction, serum NGAL was the only significant variable. We used ROC analysis to shed light on how well the examined indicators predicted sepsis mortality.

**Table 3:** Simple logistic regression analysis for prediction of mortality OR: Odds ratio; CI: Confidence interval; PRISM III: Pediatric Risk of Mortality III Score; PT: Prothrombin time; PT/INR: Prothrombin time/international normalized ratio; Cr: Creatinine; NGAL: Neutrophil gelatinase-associated lipocalin; p-value: The statistical significance level, with * indicating a significant value (usually P<0.05).

Predictors (Independent Variables)	Mortality (Dependent Variable)	OR (95% CI)	p-value
PRISM III	0.927	(0.858 - 1.003)	0.058
Multi-organ Failure	0.37	(0.109 - 1.25)	0.11
Severe Sepsis	2.364	(0.833 - 6.708)	0.106
PT	1.219	(0.99 - 1.5)	0.061
PT/INR	0.11	(0.013 - 0.911)	0.041*
Urea	1.029	(1.002 - 1.056)	0.033*
Cr	4.733	(1.292 - 17.34)	0.019*
Urea/Cr Ratio	0.985	(0.96 - 1.011)	0.259
NGAL	0.997	(0.995 - 1)	0.025*

**Table 4:** Multiple stepwise logistic regression analysis for prediction of mortality AOR: Adjusted odds ratio; CI: Confidence interval; NGAL: Neutrophil gelatinase-associated lipocalin; PT/INR: Prothrombin time/international normalized ratio; Cr: Creatinine; p-value: Indicates the level of statistical significance, where * denotes a significant p-value (usually P<0.05).

Predictors (Independent Variables)	Mortality (Dependent Variable)	AOR (95% CI)	p-value
NGAL	1.006	(1.000 - 1.012)	0.026*
PT/INR	0.261	(0.004 - 15.70)	0.520
Urea	0.961	(0.895 - 1.031)	0.276
Cr	4.984	(0.302 - 82.10)	0.261

**Table 5:** ROC analysis for mortality prediction Sensitivity: The ability of the test to correctly identify those with the disease (true positive rate). Specificity: The ability of the test to correctly identify those without the disease (true negative rate). Cut-off point: The value above or below which a test result is considered positive. NGAL: Neutrophil gelatinase-associated lipocalin; ROC: Receiver operator characteristic; PRISM III: Pediatric Risk of Mortality III Score; Cr: Creatinine; AUC: Area under the curve, a measure of the accuracy of a diagnostic test, CI: Confidence interval; P-value: Indicates the level of statistical significance, with * denoting a significant value (typically P<0.05).

Marker	AUC	95% CI	Sensitivity	Specificity	Cut-off Point	p-value
NGAL	0.691	0.574 - 0.793	100%	35%	> 990 mg/dl	0.003*
PRISM III	0.64	0.502 - 0.78	83.3%	42.9%	> 18	0.048*
Urea/Cr	0.62	0.48 - 0.875	55.6%	71.4%	< 55	0.075

**Figure 1:**
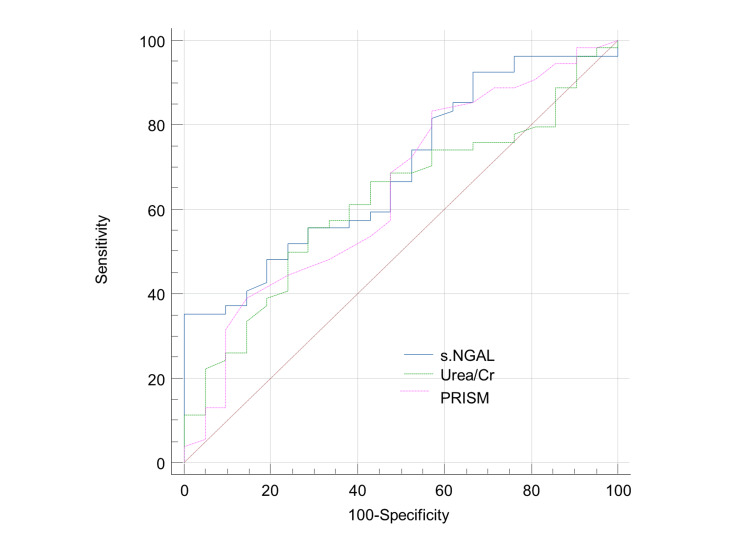
ROC curve for prediction of mortality of sepsis in children This figure shows that NGAL was the most accurate for the prediction of complications at the optimal cut-off point of 990 mg/dl, with a sensitivity of 100% and specificity of 35%, followed by PRISM at cut-off point 18 with a sensitivity of 83.3% and specificity of 42.9% and urea/Cr ratio at cut-off point 55 with a sensitivity of 55.6% and specificity of 71.4%. ROC: Receiver operator characteristic; Cr: Creatinine; NGAL: Neutrophil gelatinase-associated lipocalin.

Table 5 and Figure 1 show that, when it comes to predicting mortality at the optimal cutoff point 990, the most accurate method was NGAL with a sensitivity of 100% and specificity of 35%. PRISM came in second with a sensitivity of 83.2% and specificity of 42.9% at cutoff point 18, and the urea/Cr ratio came in third with a sensitivity of 55.5% and specificity of 71.4% at cutoff point 55 pmol/l.

## Discussion

Our research presents a prospective case-control study conducted between September 2022 and March 2023 on 75 sepsis patients recruited from the PICU. 

The study aimed to evaluate the correlation between serum NGAL and the risk of sepsis-related mortality in PICU patients. It compared serum NGAL to the PRISM III score, a widely used clinical sepsis score for predicting critical illness mortality in children, as well as to other easily calculated laboratory ratios, such as the urea/Cr ratio and the PT/INR ratio, which are routinely sampled in all children admitted to the intensive care unit with suspicion of sepsis.

Children with multi-organ failure had a greater mortality rate (38.9% in those who did not survive compared to 19% in those who did) in our research. Additionally, we found that NGAL serum levels were significantly greater in the non-survivor group compared to the survivor group.

We found similar results to Saleh et al. who found that severely ill infants with fatal outcomes had significantly higher NGAL levels at admission [26]. Moreover, NGAL levels were higher in the non-survivor group compared to the survivor group, according to the research of Kümpers et al. [28]. Patients who do not make it out of the intensive care unit have far higher amounts of NGAL in their plasma and urine when they are admitted compared to children who do survive, according to Ridder et al. [29].

One common measure used to predict patient mortality in PICUs is the PRISM III score. A large body of research has shown that it is quite sensitive and specific for determining the mortality rate of hospitalized patients [30,31]. No statistically significant difference was seen when we compared the scores of survivors with non-survivors. In line with this, Alkhalifa et al. [32] examined PRISM III's performance at a tertiary medical center in Saudi Arabia and found that, when compared to reported mortality, it performed poorly, especially in patients with respiratory disorders.

We observed that blood urea and Cr levels were significantly higher in the non-survivors compared to survivors when we investigated additional laboratory data typically taken from PICU-admitted patients with sepsis. Nonetheless, these laboratory results were not validated as reliable sepsis indicators according to several logistic regression studies.

Acute pancreatitis, bone marrow transplants, acute and chronic heart failure, and other conditions are recognized to have urea as a risk factor for death. Patients in critical care also have urea in their overall severity score [33-36]. The glomerular filtration rate is inversely proportional to Cr concentration changes. A rise in serum Cr concentration, indicative of impaired kidney function, occurs when glomerular filtration capacity declines. Even so, this metric isn't very sensitive, thus any increase in it indicates that kidney function is substantially damaged [37]. A number of variables affect the amounts of urea and Cr [15,16]. Predictions based on urea or Cr alone may not be comprehensive since urea is not a particular marker of renal insufficiency.

Prior research has shown that the urea/Cr ratio is a predictor of acute renal damage and the prognosis of patients with acute heart failure [38]. Shiba et al. demonstrated that a survival prognosis for acute heart failure patients was bad when the urea/Cr ratio was equal to or more than 22 [39]. Urea levels may rise if the intestines absorb more urea or if catabolism speeds up due to gastrointestinal hemorrhage. This indicates that a more serious situation is indicated by a greater urea/Cr ratio. A greater urea/Cr ratio was associated with an increased risk of death in septic shock patients according to Han et al. (2021). However, we found no statistically significant difference between the non-survivors and survivors in our research; this was supported by the logistic regression analysis, which again yielded an inconsequential result.

The elevated urea/Cr ratio is more likely the result of a long-term health issue than sudden low blood volume causing prerenal azotemia, as the same individuals had the greatest ratio a year before they presented to the emergency department and when they were discharged from the hospital. [40,41]. It is not possible to determine the degree of dehydration induced by elevated urea/Cr levels without knowing the patient's hydration state prior to admission. The urea/Cr ratio will also rise when blood urea levels are high (from low-grade inflammation or an unhealthy diet) and serum Cr levels are low (from a lack of muscle mass) [42-44].

In terms of coagulation indicators, there was a notable disparity between the two groups with respect to PT, with larger amounts seen in the group that did not survive. The logistic regression study, however, demonstrates that PT is not a reliable predictor of death for these individuals. Our results are in line with those of van Vught et al. who also found no correlation between admission PT and fatality rates among patients with sepsis. The fact that this distinction has nothing to do with sepsis and everything to do with liver failure could explain it [45].

Simple regression analysis linked sepsis to non-survivors with higher rates of multi-organ failure, who also had the highest PT/INR ratios. In addition, Xiang et al. found that the group with the worst prognosis due to multiple organ failure had a greater INR [46]. It is necessary to restore this link in the regression analysis, however. Additionally, in 66 patients diagnosed with sepsis or septic shock, Liu et al. discovered that an elevated INR was linked to an elevated risk of 28-day all-cause death [47]. The PT/INR is a modest diagnostic tool for septic shock and sepsis according to Schupp et al. [48].

To predict mortality, an ROC curves model was used for the markers that were investigated. With a cutoff value of >90%, sensitivity of 100%, and specificity of 35%, NGAL stood up as the most accurate predictive biomarker. Our data suggests that NGAL stands on its own as a mortality predictor. The correlation between NGAL plasma levels, intensive care unit admission, and in-hospital mortality was also investigated by Min et al. in a study of 241 pneumonia patients hospitalized. Levels of NGAL were significantly higher among patients admitted to the intensive care unit compared to those who were released. Also, NGAL levels were more likely to be elevated in patients who did not make it through the procedure than in those who did [49].

Children admitted to the PICU due to sepsis may have elevated levels. There are better ways to predict severity and survival rate than NGAL levels assessed during the first hour of arrival. Since they are more often associated with organ failure than sepsis, urea/Cr and PT/INR ratios cannot be used as standalone mortality predictors. Although the pathophysiology of multi-organ failure is more prevalent in non-survivors than survivors, regression analysis shows that this reversible state is not a predictor of death. When it comes to protecting microorganisms, innate immunity relies heavily on NGAL. In cases of sepsis linked to organ failure, it may serve as a targeted indicator for the body's defensive mechanisms.

The maturation of neutrophils relies on the NGAL protein. When neutrophils reach the last stage of their maturation, secretion of the NGAL protein is the last step in removing the protein from the cell [50]. In an iron-limited environment, the circulating NGAL protein is bound to the siderophore iron, which restricts bacterial growth. Several cytokines and growth factors, including interleukin 1 (IL-1), interleukin 17 (IL-17), interleukin 22 (IL-22), insulin-like growth factor-1, tumor growth factor-beta (TGFβ), and TNFα, have the ability to stimulate NGAL expression, which aids in its protective role [50,51]. Mice engineered to lack both copies of the NGAL gene were shown to be more susceptible to bacterial infections and sepsis compared to their wild-type counterparts [52,53]. As a result, NGAL plays a significant role in the immune response to bacterial infections.

Due to its limited sample size and the fact that it was carried out in a single hospital, this study has several limitations that may need more research with a broader patient population.

## Conclusions

The study concludes that serum NGAL levels at PICU admission are significantly associated with increased mortality in children with sepsis and may serve as a more sensitive predictor of mortality than the widely used PRISM III score, urea/Cr ratio, and PT/INR. While the PRISM III score did not show a significant difference between survivors and non-survivors, NGAL demonstrated higher sensitivity in predicting outcomes, suggesting its potential role in early diagnosis and intervention for sepsis-related mortality in pediatric patients. However, larger multi-center studies are required to confirm these findings and assess the broader clinical utility of NGAL.
